# An *in vitro* testing strategy towards mimicking the inhalation of high aspect ratio nanoparticles

**DOI:** 10.1186/s12989-014-0040-x

**Published:** 2014-09-23

**Authors:** Carola Endes, Otmar Schmid, Calum Kinnear, Silvana Mueller, Sandra Camarero-Espinosa, Dimitri Vanhecke, E Johan Foster, Alke Petri-Fink, Barbara Rothen-Rutishauser, Christoph Weder, Martin JD Clift

**Affiliations:** BioNanomaterials, Adolphe Merkle Institute, University of Fribourg, Chemin des Verdiers 4, CH-1700 Fribourg, Switzerland; Comprehensive Pneumology Centre, Institute of Lung Biology and Disease, Helmholtz Zentrum Muenchen, Ingolstädter Landstraße 1, 85764 Neuherberg, Germany; Polymer Chemistry and Materials, Adolphe Merkle Institute, University of Fribourg, Chemin des Verdiers 4, CH-1700 Fribourg, Switzerland; Department of Chemistry, University of Fribourg, Fribourg, Switzerland

**Keywords:** High aspect ratio nanoparticles, Air liquid interface, In vitro, Inhalation, Characterisation, Alternative testing strategies, Cellulose nanocrystals

## Abstract

**Background:**

The challenge remains to reliably mimic human exposure to high aspect ratio nanoparticles (HARN) via inhalation. Sophisticated, multi-cellular *in vitro* models are a particular advantageous solution to this issue, especially when considering the need to provide realistic and efficient alternatives to invasive animal experimentation for HARN hazard assessment. By incorporating a systematic test-bed of material characterisation techniques, a specific air-liquid cell exposure system with real-time monitoring of the cell-delivered HARN dose in addition to key biochemical endpoints, here we demonstrate a successful approach towards investigation of the hazard of HARN aerosols *in vitro*.

**Methods:**

Cellulose nanocrystals (CNCs) derived from cotton and tunicates, with differing aspect ratios (~9 and ~80), were employed as model HARN samples. Specifically, well-dispersed and characterised CNC suspensions were aerosolised using an “Air Liquid Interface Cell Exposure System” (ALICE) at realistic, cell-delivered concentrations ranging from 0.14 to 1.57 μg/cm^2^. The biological impact (cytotoxicity, oxidative stress levels and pro-inflammatory effects) of each HARN sample was then assessed using a 3D multi-cellular *in vitro* model of the human epithelial airway barrier at the air liquid interface (ALI) 24 hours post-exposure. Additionally, the testing strategy was validated using both crystalline quartz (DQ12) as a positive particulate control in the ALICE system and long fibre amosite asbestos (LFA) to confirm the susceptibility of the *in vitro* model to a fibrous insult.

**Results:**

A rapid (≤4 min), controlled nebulisation of CNC suspensions enabled a dose-controlled and spatially homogeneous CNC deposition onto cells cultured under ALI conditions. Real-time monitoring of the cell-delivered CNC dose with a quartz crystal microbalance was accomplished. Independent of CNC aspect ratio, no significant cytotoxicity (*p* > 0.05), induction of oxidative stress, or (pro)-inflammatory responses were observed up to the highest concentration of 1.57 μg/cm^2^. Both DQ12 and LFA elicited a significant (*p* < 0.05) pro-inflammatory response at sub-lethal concentrations *in vitro*.

**Conclusion:**

In summary, whilst the present study highlights the benign nature of CNCs, it is the advanced technological and mechanistic approach presented that allows for a state of the art testing strategy to realistically and efficiently determine the *in vitro* hazard concerning inhalation exposure of HARN.

**Electronic supplementary material:**

The online version of this article (doi:10.1186/s12989-014-0040-x) contains supplementary material, which is available to authorized users.

## Introduction

Commercial production of a diverse range of high aspect ratio nanoparticles (HARN; aspect ratio ≥3) is rapidly increasing, with an inevitability that they will be readily exposed to humans [1,2]. Thus, inhalation of airborne HARN is a realistic scenario, posing a potential risk towards human health [3–5] within either an occupational or consumer setting [6]. Due to their low aerodynamic diameter, HARN may enter the deepest regions of the human lung freely [3]. Understanding the potential for HARN to cause adverse health effects is limited at this moment in time due to a lack of test systems that can efficiently mimic human exposure to HARN aerosols and provide insight into the mechanistic HARN-cell interaction [3]. In this context, as well as the research principles of the 3R (refine, reduce and replace) [7], there is an urgent requirement for realistic and reliable testing strategies that may be used to gain an intrinsic understanding of how HARN could affect the lung at the cellular level [8], as well as provide an alternative to invasive animal experimentation [9]. It must also be highlighted that *in vivo* approaches can lack understanding of species specificity and cellular mechanics, suggesting that information gained from such experimental approaches may not necessarily be predictive of human exposure effects to HARN [10]. On the other hand, well-characterised and anatomically correct multi-cellular *in vitro* models offer an ideal platform to systematically elucidate both organ specificity and localised cellular influences for any potentially harmful effects that could be induced by HARN [11].

When considering the human lung *in vitro* the use of air liquid interface cell cultures represent a more realistic perspective [12], especially when compared to submerged exposures, due to increased exposure specificity and the absence of suspension artifacts [13]. Currently, no air-liquid exposure systems have been shown to reliably and efficiently nebulise HARN for *in vitro* toxicology research. Instead, such *in vitro* systems have focused on the nebulisation effects of spherical (nano)particles [14–17]. Most systems that have attempted to nebulise HARN however, have been specific to animal-based experimental strategies [18–24]. Thus, a new and advanced *in vitro* testing strategy to investigate the risk of HARN from a perspective of inhalation exposure is presented, using cellulose nanocrystals (CNCs) as a model. With similar beneficial mechanical and physical properties to those of carbon nanotubes [25,26], CNCs are on the verge of mass production [27], and thus pose a potentially heightened risk for human exposure, notably *via* inhalation.

The afore-mentioned testing strategy was achieved by systematically combining three cornerstones of nanotoxicology research. A thorough material characterisation was performed to gain an intrinsic understanding of the material prior to, and following aerosolisation. Aerosols were produced to mimic HARN inhalation using the “Air Liquid Interface Cell Exposure System” (ALICE), as described by Lenz *et al*. [16] for spherical nanoparticles, thereby establishing the efficient nebulisation and real-time monitoring of well-dispersed HARN with the ALICE. These components were complemented by the use of a sophisticated 3D *in vitro* model of the epithelial airway barrier [11] to assess the systems’ applicability for HARN hazard assessment *in vitro*. Furthermore, to confirm the sensitivity of the biological system within this experimental set-up, crystalline quartz (DQ12) and long fibre amosite asbestos (LFA), both known for their pathogenic effects, were included as positive ‘crystalline particle’ and ‘fibrous particle’, respectively. Combined, this satisfied a number of current knowledge gaps within the field regarding applicable, alternative *in vitro* testing strategies.

## Results

### Characteristics of cellulose nanocrystals

**Figure 1:**
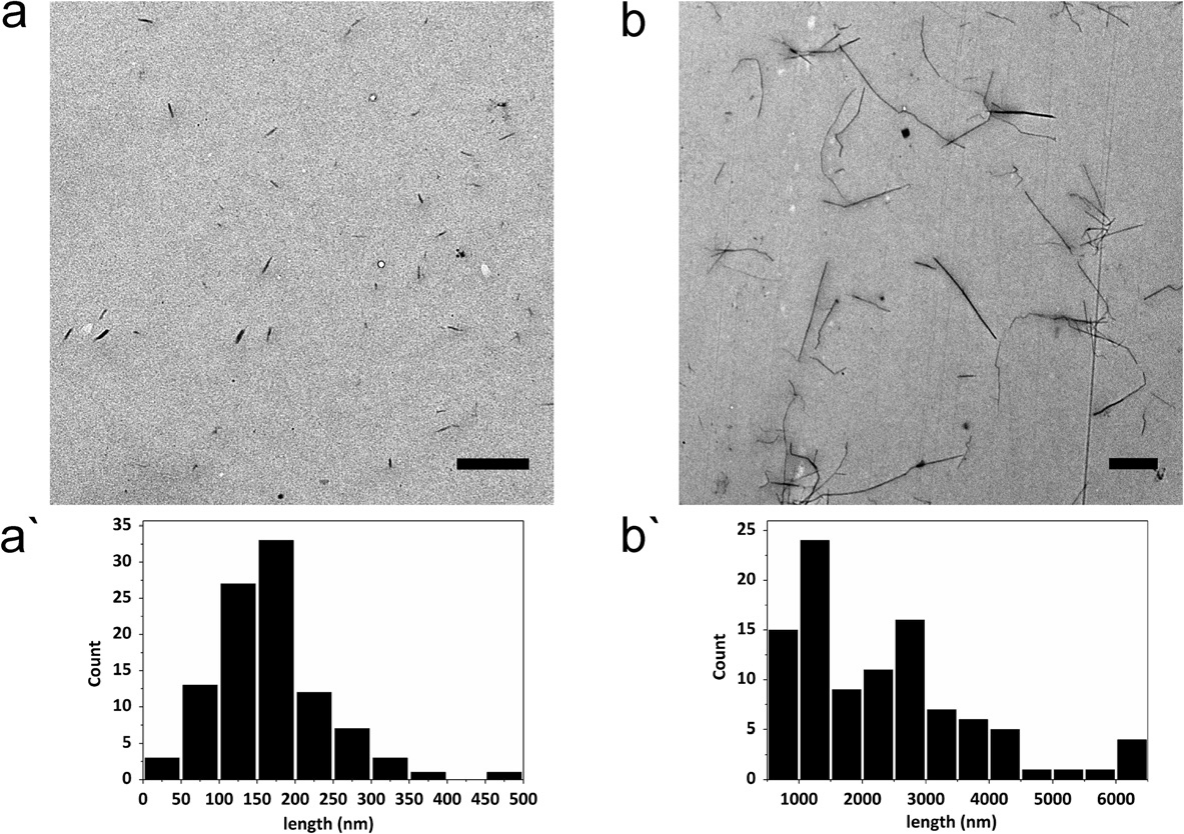
**Morphology of CNCs in stock suspension**. Transmission electron microscopy images of c-CNCs **(a)** and t-CNCs **(b)** after dispersion in ultrapure water. Histograms show the size distribution as counts (y-axis) and length (nm) (x-axis) of c-CNCs **(a`)** and t-CNCs **(b`)**. Scale bars represent 1 μm. Dimensions were derived from 100 individual measurements of 3 individual nebulisations (n = 3).

Detailed physico-chemical characterisation of both CNC types and additional information, also to the control materials, is summarised in Tables 1 (CNCs) and 2 (DQ12 and LFA). Following extraction, dispersion by sonication in ultrapure, deionised water led to rod-shaped fibers with a common size distribution, as determined from transmission electron microscopy (TEM), with averages of 170 ± 72 nm in length and 19 ± 7 nm in width for c-CNCs (Figure 1a) and longer fibers of 2.3 ± 1.4 μm in length and 31 ± 7 nm in width for t-CNCs (Figure 1b). The nanocrystals displayed the typical chemical composition of pure cellulose, with trace amounts of sulfur introduced solely by the isolation process. As expected [25,28], both types of CNCs formed stable suspensions after dispersion in ultrapure water and following strong ultrasound sonication for 1 h. Further to this, depolarized dynamic light scattering (DDLS) showed no significant changes in rotational or translational diffusion co-efficients of the suspensions over a 24 h period, indicating that the suspensions were stable under these conditions [29] (Table 1). Visual examination of the suspension after two weeks (Additional file 1: Figure S1) further supported the stability of the CNC as shown by DDLS, suggesting that suspensions of this nature can remain stable in this environment for up to several weeks, similarly in the presence of NaCl.

**Table 1:** **Physico-chemical characteristics of CNCs derived from cotton (c-CNC) or tunicates (t-CNC)**

	**Dimension** ^**a**^ **(nm)**	**Aspect ratio** ^**a**^	**Chemical composition** ^**b**^ **(%)**	**Surface charge** ^**c**^	**Stiffness**	**Stability** ^**d**^	
				**(mmol SO**_**4**_^**−**^ **/kg)**	**(GPa)**	**D**_**T**_ **×10** ^**−8**^ **(cm** ^**2**^ **/s)** ^**e**^	**D**_**R**_ **(s** ^**−1**^ **)** ^**f**^
**c-CNC**	170 ± 72 (l) x	9.2 ± 3.7	(C)41.45 ± 3.02^**g**^	56 ± 4.9	~105 [57]	(0 h) 2.81 ± 0.32	(0 h) 64.5 ± 4.0
	19 ± 7 (w)		(H)6.03 ± 0.48^**h**^			(24 h) 3.11 ± 0.14	(24 h) 67.5 ± 8.8
			(S)0.18 ± 0.03				
**t-CNC**	2300 ± 1400 (l)	80 ± 21	(C)39.88 ± 0.66^**g**^	133 ± 2.4	~143 [58]	(0 h) 2.24 ± 0.23	(0 h) 42.5 ± 13.0
	x 31 ± 7 (w)		(H)5.73 ± 0.06^**h**^			(24 h) 2.36 ± 0.32	(24 h) 34.1 ± 8.8
			(S)0.47 ± 0.05				

^a^Transmission electron microscopy, ^b^Elemental analysis, ^c^Titration, ^d^Depolarised dynamic light scattering, ^e^Translational diffusion coefficient, ^f^Rotational diffusion coefficient; theoretical values for pure cellulose ^g^(C) 44.31% and ^h^(H) 6.51%.

Data is presented as the mean ± standard deviation (SD), except for depolarised dynamic light scattering (DDLS), where standard error of the regression analysis is given (SE).

**Table 2:** **Physico-chemical characteristics of DQ12** [56] **and LFA** [5,45,46]

	**Dimension (μm)**	**Geometric aspect ratio**	**Chemical composition (%)**	**Stiffness (10** ^**3**^ **kg/cm** ^**2**^ **)**
**DQ12**	0.7-6	-	crystalline SiO_2_ 87	-
			amorphous SiO_2_	
			Na_2_O, K_2_O, LiO2, Al, K, Ca, Ti, Fe	
**LFA**	5.37 (l) × 0.40 (w)	8.8*	SiO_2_ 49-52*	1620 [40]
			FeO 35-40	
	50.36% >15 (l)^#^		Fe_2_O_3_ 0-5	
	35.25% > 20 (l)^#^		MgO 5-7	
			Al_2_O_3_, CaO, Na^2^O, N^2^O^+^	

*Deduced from the geometric mean length (3.1 μm) and width (0.35 μm) of LFA [46].

^#^Key size ranges.

### Nebulisation of CNCs/HARN

In the present study, the ALICE has been used for the first time to establish the nebulisation of HARN, using CNCs as an example, to provide a basis for realistic inhalation hazard assessment of HARN *in vitro*. To confirm cloud production efficiency, cloud production rate was monitored by nebulising 1 mL of c-CNC and t-CNC suspensions, respectively. The addition of ions, in this case 500 μM NaCl, to the CNC suspensions permitted ideal nebulisation of CNCs, while concomitantly improving and stabilising the output rates significantly without interfering with quantification of CNC deposition (Additional file 2). This format ensured a repeatable and efficient dense cloud production, a controlled CNC delivery of all c-CNC or t-CNC test concentrations (0.1, 0.5, 1.0 mg/mL), as well as the negative control (500 μM NaCl) (Additional file 3: Table S1). The mean output rate for each HARN material closely matched the cloud production of zinc oxide nanoparticles and a basic salt solution (90–150 s for nebulisation of 1 mL), as previously reported by Lenz *et al.* [16].

### Deposition characterisation

**Figure 2:**
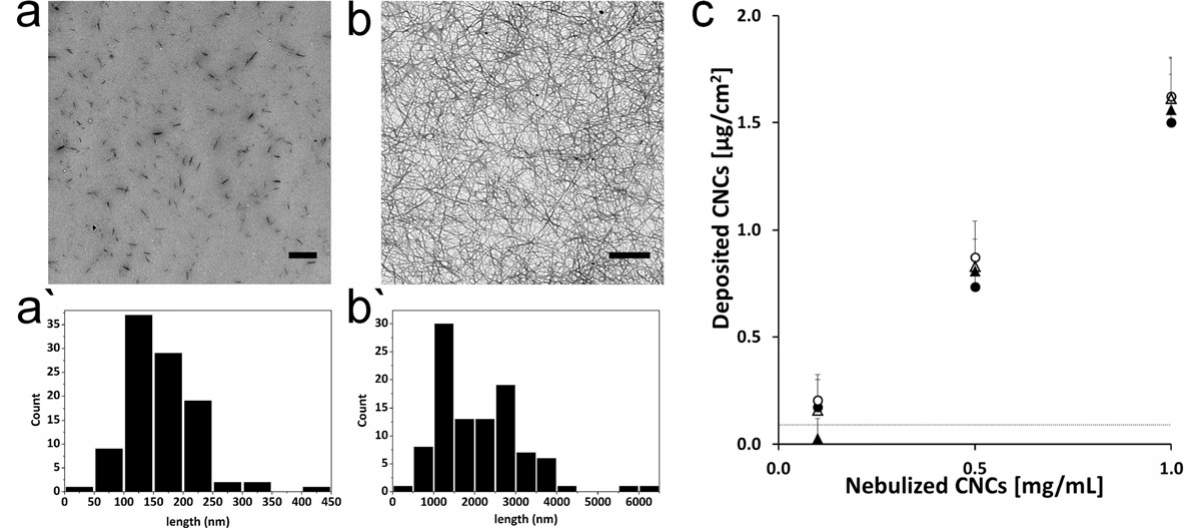
**Morphology and quantification of deposited CNC aerosols.** Transmission electron microscopy images of 1 mL nebulised c-CNCs **(a)** and t-CNCs (both 0.5 mg/mL) **(b)** deposited on pre-coated, protein rich copper grids using the ALICE system resulting in a deposited dose of 0.81 ± 0.03 μg/cm^2^. Histograms show the size distribution as counts (y-axis) and length (nm) (x-axis) of c-CNCs **(a`)** and t-CNCs **(b`)**. Dimensions are measured from pictures derived from nebulisations leading to mostly individually deposited fibers (c-CNCs 0.5 and 0.1 mg/mL; t-CNCs 0.1 mg/mL). Average dimensions were calculated from 100 individual measurements of 3 individual nebulisations (n = 3). Quantification of deposited c-CNCs and t-CNCs after nebulisation at 0.1, 0.5 and 1 mg/mL was performed using two independent methods (n = 3) **(c)**. Dots (○) represent results for c-CNCs, triangles (Δ) t-CNCs; filled symbols = QCM data, empty symbols = Anthrone data. The dotted line (…..) displays the detection limit of the QCM (90 ng/cm^2^). Scale bars represent 1 μm.

By exposing protein pre-coated copper grids placed at the bottom of the ALICE, the morphology of deposited CNCs was investigated *via* TEM (Figures 2a and 2b, and Additional file 4: Figure S2 a-d). Quantitative analysis of the TEM images revealed that the deposited c-CNCs had a length of 161 ± 61 nm and a diameter of 16 ± 5 nm, whereas t-CNCs had a length of 2.1 ± 1.0 μm and a width of 20 ± 5 nm (aspect ratios: c-CNCs 11 ± 5; t-CNCs 108 ± 57). To quantify the deposition of the two nanocrystal types on the bottom of the ALICE, two independent methods were applied; (i) an integrated quartz crystal microbalance (QCM) for real-time monitoring of cell-delivered dose and (ii) the system independent anthrone assay. Results showed a controlled, dose-dependent, and repeatable deposition of both CNCs types when nebulising 1 mL suspension (Figure 2c, filled spheres and triangles). The anthrone assay (Figure 2c, empty spheres and rectangles) validated the results of the QCM measurements as a useful tool to detect the cell-delivered HARN dose. Most importantly, the anthrone assay as a reference method supported the findings of the QCM at the lowest deposited dose, which lay near to the QCM detection limit (90 ng/cm^2^) (filled rectangle at 0.1 mg/mL t-CNCs, Figure 2c). Average deposited doses of both materials and methods were 0.14 ± 0.04, 0.81 ± 0.03 and 1.57 ± 0.03 μg/cm^2^. The negative control (500 μM NaCl) was below the detection limit in both methods. A deposited dose of 0.23 ± 0.08 μg/cm^2^ was recorded for DQ12. The deposition efficiency for nebulizing CNCs with the ALICE system was calculated as 67 ± 8% for c-CNCs and 64 ± 1% for t-CNCs, which is in agreement with the deposition efficiency of spherical nanoparticles (~57 ± 7%), as previously reported [16]. Additionally, the anthrone assay [30] quantitatively confirmed the deposition pattern of c-CNCs, as well as t-CNCs, within the ALICE to be highly uniform, supporting the qualitative impression formed for the deposition of CNCs with the ALICE *via* TEM (Figures 2a and 2b, Additional file 4: Figure S2 a-d), when comparing the deposited CNCs in each of the six individual wells of a trans-well plate for cell culture (Additional file 5: Figure S3). This mentioned, visual examination of deposited CNCs on exposed pre-coated, protein rich copper grids demonstrated that the nebulisation process, particularly the vibrating membrane and its pore-size, did not induce any rupture or fracture upon the fiber-shaped nanocrystals (Figures 2a and 2b and Additional file 4: Figure S2 a-d).

### Hazard assessment of CNC aerosols

**Figure 3:**
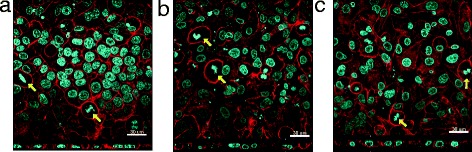
**Cell morphology of CNC exposed cell cultures.** Confocal laser scanning microscopy analyzes visualising the actin cytoskeleton (red) and the nuclei (cyan) of control **(a)** or 1.57 μg/cm^2^ of either c-CNCs **(b)** or t-CNC **(c)** exposed cells. The images represent xy- and xz-projections. Yellow arrows indicate cells undergoing cell division. Scale bars are 30 μm.

**Figure 4:**
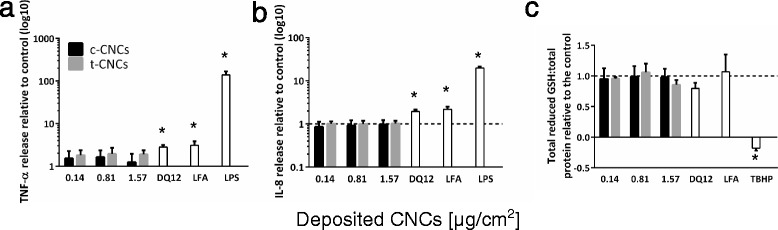
**Biochemical response of triple cell co-culture system following CNC exposure. (a)** Tumor necrosis factor α (TNF-α) release, **(b)** Interleukin 8 (IL-8) release and **(c)** oxidative stress status of the *in vitro* triple cell co-culture model after exposure to c-CNCs (black) or t-CNCs (grey) to the three test concentrations (n = 3; +SEM). The respective controls are shown in white; DQ12 [0.23 ± 0.08 μg/cm2], LFA [100 μL of 0.05 mg/mL], LPS [100 μL of 1 μg/mL] and TBHP [250 μL of 100 mM]. Dashed lines (--- ) represent the level of the negative control. Data is presented as the fold increase relative to the negative control. TNF-α and IL-8 data is expressed as a logarithmic scale (y-axis). * equals *p* < 0.05.

For each CNC material, at all tested concentrations no significant cytotoxicity was observed, as measured by lactate dehydrogenase (LDH) release (Additional file 6: Figure S4). Similar findings were also evident for nebulised DQ12 ([0.23 ± 0.08 μg/cm^2^]) and the positive fibrous control LFA (Table 2). In the case of LFA, 100 μl of the suspension at 0.05 mg/mL was dropped onto the apical side of the *in vitro* model at the air liquid interface, (referred to as pseudo-ALI). These findings were subsequently confirmed by the observation that no alteration to cell morphology occurred after CNC exposure, as analysed by confocal laser scanning microscopy, when compared to the negative control. The cells formed a tight monolayer and also cell division was regularly observed for all conditions tested (Figure 3, cell division is denoted by yellow arrows). It also could be shown that DQ12 and LFA exposed cells did not undergo any cellular changes and in addition no cytotoxicity was observed compared to the negative control (Additional file 6: Figure S4 and Additional file 7: Figure S5). Subsequent assessment of the potential sub-lethal effects of aerosolised c-CNCs and t-CNCs analysed the oxidative stress status of the 3D cell model by assessment of the total amount of reduced glutathione (GSH), a key marker for oxidative stress [31], and the (pro-)inflammatory response *via* cytokine (tumor necrosis factor(TNF)-α) and chemokine (interleukin(IL)-8) secretion (Figure 4). At all concentrations tested no significant changes (*p* > 0.05) to the oxidative stress status of the cell, or any mediated (pro-)inflammatory response were observed for either CNC type. In contrast, DQ12 and LFA elicited a notable, statistically significant (*p* < 0.05) release of TNF-α and IL-8 after 24 h. Interestingly, DQ12 also showed a slight depletion, although not significant (*p* > 0.05) in the total amount of reduced GSH at this time point, most likely due to the enhanced inflammatory response. LFA was not found to elucidate an oxidative stress response within the triple cell co-culture at the pseudo-ALI.

## Discussion

In the current study a successful approach to a next-level testing strategy for *in vitro* inhalation toxicology research for HARN is presented. The ALICE, as a reliable nebulisation platform for spherical particles [16], proved to be applicable for the aerosolisation of HARN, as shown here with CNCs either isolated from cotton (c-CNCs) [32] or tunicates (t-CNCs) [33,34] (Table 1). Prolongation of output rates by formed air bubbles or CNC-clusters were successfully overcome by gaining a thorough understanding of the materials’ physico-chemical characteristics, especially their colloidal stability, as a pre-requisite for controlled nebulisation under the applied conditions. Material characterisation before and after the nebulisation process, as well as potential damage to CNC morphology by the vibrating membrane of the nebuliser was successfully excluded [35]. The deposition of CNCs via the ALICE system was shown to fulfill all cornerstones when compared to spherical particles [16], namely controllability, dose dependency, spatial homogeneity and deposition efficiency throughout all tested CNC concentrations and lengths. In this context, the QCM was used as a real-time monitoring tool for the cell-delivered CNC dose, as validated with an off-line reference method (anthrone assay), giving important implications for future HARN studies at the ALI. The applicability of the system for HARN aerosolisation has been further demonstrated via the nebulisation of multi-walled carbon nanotubes (unpublished data). Hence, the ALICE system can be considered as a highly suitable method for conducting realistic *in vitro* inhalation hazard assessment of HARN.

The results of the hazard assessment presented in this study highlight the benign nature of c-CNCs and t-CNCs and are in accordance with previous studies [36,37]. Both studies have shown CNCs, at lower doses (Kovacs *et al.* 0.03–10 g/L [36]; Peireira *et al.* 0.02–100 μg/mL [37]), to cause a limited adverse effect upon different biological systems (*e.g.* Rainbow trout, *Daphnia magna*, *Vibrio fischeri*, *Pseudokirchneriella subcapitata*, fibroblasts) following exposure. In the present study, it must be considered that all exposure concentrations used can be defined as “overload” situations. Despite this, they can be justified by extrapolating to the OSHA permissive exposure limits (PEL) for cotton dust in cotton weaving industry workplaces (200 – 750 μg/m^3^ 8 h time weight average) [38]. Following the considerations by Lenz *et al*., presuming 50% nanofibre deposition in the alveoli due to an unclear quantitative background and 50% clearance in the case of a healthy lung, together with a PEL of 750 μg/m^3^, the lowest exposure concentration tested in the present study corresponds to an equivalent pulmonary CNC dose received by a worker after 15 working weeks, up to three working years (at the highest deposited dose used) [16]. In this regard, despite further research being necessary, within the acute time period tested here the stiffness/rigidity of the CNCs does not appear to influence the biochemical cascade(s) reflected by the measured endpoints. This finding is in contrast to previous findings for carbon nanotubes and asbestos fibres which, in the same range of rigidity [39,40], elicit heightened adverse biological effects [3]. In contrast to the presented results here, Clift *et al*. [41] showed previously that c-CNCs delivered by suspension to the same *in vitro* model can trigger a dose-dependent cytotoxicity and (pro-) inflammatory response. The differences in results highlight the influence of the exposure method on the characteristics of the material which can strongly influence the biological response by c-CNCs. Such influences can be attributed to the interaction of the CNCs with the components of the cell culture medium used, and most notably the formation of a protein coating [13]. The lower secretion of cytokines in the ALI cell system could also be influenced by the lower diffusion rate due to absence of media in the upper well in contrast to suspension exposure scenarios.

To validate the testing strategy in terms of biological sensitivity, a positive particulate aerosol control, DQ12, and a positive fibre control (LFA) at the pseudo-ALI (100 μl of 0.05 mg/mL LFA suspension applied to the apical side (Table 2), were used. DQ12 showed the applicability of ALICE nebulisations of a particulate agent causing (pro-)inflammatory reactions in combination with the *in vitro* triple cell co-culture model at the air-liquid interface, whilst LFA, exposed using pseudo-ALI exposures, further demonstrated the susceptibility of the multi-cellular model to a fibrous insult (Figure 4; Table 2). DQ12 and LFA elicited a minor but biologically relevant TNF-α release, inducing a strong and significant IL-8 release (*p* < 0.05) after 24 h indicating that (pro-)inflammatory responses can be triggered by known, classical particulate and fibrous inflammogens, such as DQ12 [42–44] and LFA [45,46]. The results presented here therefore validate the testing strategy in combination with the co-culture model for the detection of respiratory toxicity of high aspect ratio nanoparticles and fibres *in vitro*.

Finally, the notable lack of biochemical effects observed upon acute exposure to c-CNCs and t-CNCs, respectively, does not necessarily indicate that these organic HARN do not elicit biochemical reactions within the multi-cellular model in a chronic study. Predominantly, despite the absence of a (pro-)inflammatory response in the acute scenario, fiber-associated responses like pro-fibrogenic effects need to be taken into consideration within a chronic time period due to prolonged continuous, or repeated exposure to these HARN. Further to these aspects, additional ongoing research also focuses on the biodurability of these materials as a key cornerstone for their potential long-term toxicity. Additionally, the small decrease of antioxidants (GSH) in the highest exposed dose of t-CNCs (1.57 ± 0.03 μg/cm^2^; Figure 4c) could hint at oxidative stress caused by enhanced reactive oxygen species formation because of nanocrystal interaction with mitochondria or membrane-bound NADPH oxidases, with an absent (pro-)inflammatory response (Figure 4a and 4b) [47]. Findings by Peireira and colleagues [37], who measured up-regulated mRNA levels for the antioxidant protein Peroxiredoxin 1 (PRDX1) after suspension exposure of fibroblasts to high doses (2–5 mg/mL) of cotton CNCs, support these results. It is therefore necessary that further studies are conducted to elucidate the impact on the oxidative state by applying realistic doses, chronically, to a relevant target tissue.

## Conclusions

In the present study, an advanced mechanistic approach has been formulated to create a state of the art testing strategy for conducting realistic and efficient *in vitro* hazard assessment concerning the inhalation exposure of HARN. Further to this reliable and efficient outlook towards assessing the potential risk of HARN, the strategy portrayed herein can be considered as an advantageous alternative to existing *in vitro* methodologies as well as a valid tool in the refinement, reduction and replacement of animal testing. Finally, the low-toxicity potential of CNCs independent of their length could be demonstrated, supported by relevant inflammogenic particulate and fibrous positive controls.

## Methods

### Chemicals and reagents

All chemicals and reagents were purchased from Sigma-Aldrich (Switzerland), unless otherwise stated. Ultrapure, deionised water (sartorius stedim biotech, arium® 611DI; sartorius, Germany) was used throughout all experiments.

### Extraction of cotton and tunicate cellulose nanocrystals

Cotton cellulose nanocrystals (c-CNCs) were extracted from Whatman No. 1 filter paper (WhatmanTM Ltd., England) following the protocol by Capadona *et al*. [48], that is a modification of the method previously described by Dong *et al*. [32]. Briefly, Whatman filter paper was blended with ultrapure water. Subsequently, concentrated sulfuric acid (95–97%) was slowly added to the sample on ice keeping the temperature below 30°C. Hydrolysis was accomplished by stirring for 4.5 h at 50°C. After several centrifugation steps, the resulting nanocrystals were dialyzed until the solution reached a neutral pH and then subsequently sonicated for 4 h.

Tunicate cellulose nanocrystals (t-CNCs) were prepared from the species *Styela clava* as previously described [33,34]. After harvesting the sheaths of the animals, the material was cleaned by stirring in potassium hydroxide at 80°C overnight. After rinsing and bleaching the material, the above mentioned treatment with sulfuric acid for c-CNCs followed. After hydrolysis, the resulting mixture was filtrated and dialyzed to a neutral pH. Both materials were freeze-dried for storage at room temperature until required.

### Preparation of c-CNCs and t-CNCs samples

To produce homogenous suspensions for ALICE experiments, dry CNCs were weighed out to a concentration of 1 mg/mL and suspended in ultrapure water. Specifically, pre-mixed solutions were sonicated for 1 h at a frequency of 37 kHz and 100% intensity in pulse mode in a flat bottom glass flask (Duran® Schott, Germany) in an Elmasonic P30.H sonication bath (Elma®, Hans Schmidbauer GmbH&CoKG, Singen, Germany). The stock suspension of c-CNCs and t-CNCs were diluted further with ultrapure water to additional test concentrations of 0.5 and 0.1 mg/mL.

### Characterisation of cellulose nanocrystal stock suspensions

#### Elemental analysis

c-CNC and t-CNC dry powders (2–5 mg) were measured in a precision balance and placed into an universal “soft” tin container (Thermo Scientific, USA). Investigation of the elemental composition of each sample was then performed in a Flash 2000 Organic Elemental Analyzer (Thermo Scientific, USA), equipped with copper oxide filled CHNS/O columns. For analysis, only a CHNS column was used with helium as a carrier gas and oxygen as an oxidizing agent. The Elemental Analyzer was calibrated prior to each use by measuring three standards of the same 2,5-bis(5-tert-butyl-2-benzo-oxazol-2-yl) thiophene compound. Subsequently, samples of c- and t-CNCs were measured and chromatograms were analysed by the Eager Xperience computer software (Brechbühler AG, Switzerland).

### Charge density determination

Surface charge density of cellulose nanocrystals was determined by conductometric titration, as previously reported [33,49]. Cellulose nanocrystals (50 mg) were dispersed in 10 mL of 0.01 M HCl by sonication. Subsequently, the suspension was titrated with 0.01 M NaOH recording the conductivity of the suspension versus the volume of NaOH added to the suspension. For conductivity measurements, a Mettler Toledo SevenmultiTM pH-meter connected with a conductivity measurement electrode was used. To determine the charge density, the charge titration graph was divided in three parts and a linear fit was applied; (i) titration of excessive HCl, (ii) titration of sulfate groups on the surface of CNCs and (iii) addition of excess NaOH at the end of the titration. The quantity of NaOH required to titrate the sulfate groups was calculated using the intercept points, from which the charge density of CNCs was finally estimated using equation (1).

1 $$ \frac{n\left(S{O}_4^{-}\right)}{m(CNCs)}=\frac{c(NaOH)\times V(NaOH)}{m(CNC)} $$

[c(NaOH) = 0.01 mol/L (vide supra), V(NaOH) = volume of NaOH required to titrate all sulfate groups, m(CNC) = 50 mg (vide supra)]

### Stability

Based on the previous description by Lima and colleagues, the translation and rotational diffusion coefficients of c-CNCs and t-CNCs were used to assess their stability in water at a concentration of 1 mg/mL, measured using depolarised dynamic light scattering (DDLS) [29]. Analysis was performed immediately after preparation of the CNC dispersion by ultra-sonication and after a 24 h resting-period. DDLS was carried out using a Goniometer System from LS Instruments (Switzerland) using a He-Ne laser as the light source. A Glan-Thompson polarizer with an extinction ratio of >10^-8^ was placed before the detector and aligned perpendicular to the polarization of the incident light. DDLS measurements were carried out at 20°C and in the angle range of 30°-135°. The correlation function obtained at each angle was analysed *via* CONTIN. This was then plotted against the square of the scattering angle, allowing the translational diffusion co-efficient and the rotational diffusional co-efficient to be obtained from the gradient and intercept of the plot respectively.

### Transmission electron microscopy

A protein rich, aqueous suspension of CNCs stock was diluted to a final concentration of 1.5 μg/mL (c-CNCs) and 2.5 μg/mL (t-CNCs). Of each sample, 4 μL was cast onto a TEM copper grid (Plano, Germany) and air dried. For characterisation of the deposited c-CNCs and t-CNCs after nebulisation within the ALICE, pre-coated copper grids were exposed to nebulisations of the lowest concentration of CNCs leading to mostly individually deposited fibres to avoid interaction on the TEM grid for analysis of their dimensions (c-CNCs 0.5 and 0.1 mg/mL; t-CNCs 0.1 mg/mL). Representative images of both the stock solutions and deposited samples were captured using a Hitachi H-7100 (Hitachi, Japan) at 75 kV equipped with a Morada 11 MPix digital CCD camera (Olympus, Japan). The length and width of both the c-CNCs and t-CNCs were subsequently analysed manually using ImageJ software [50]. Data presented is representative of 100 individual measurements on at least 5 different micropgraphs taken from 3 individual nebulisations (n = 3) (mean ± SD).

### Nebulisation of CNCs with the “Air Liquid Interface Cell Exposure System”

#### Nebulisation procedure

The “Air Liquid Interface Cell Exposure System” (ALICE) has been described previously by Lenz *et al.* [16]. The aerosol is generated into the exposure chamber by a perforated vibrating membrane nebuliser (customised eFlow nebuliser system, PARI Pharma GmbH, Germany) [51]. For each nebulisation 1 mL of suspension was used with ranging CNC concentrations of 0.1, 0.5 and 1 mg/mL CNCs with 500 μM NaCl (NAAPREP® physiological saline, GlaxoSmithKline, France) respectively. Samples were added immediately prior to nebulisation. NaCl in the respective concentration served as a control exposure.

### Video of nebulisation

A representative visualisation of nebulisation (~15 min) with 0.5 mg/mL t-CNCs in 500 μM NaCl is shown in the Additional file 2.

### Quartz crystal microbalance

As an integrated part of the ALICE, the quantification of deposited material was monitored by a quartz crystal microbalance (QCM, detection limit 90 ng/cm^2^, AT-cut quartz, 5 MHz resonance frequency, Stanford Research Systems, USA). After each complete nebulisation process the QCM was removed from the chamber and air dried to remove residual water [16]. Due to the settling of material onto the QCM, the frequency of the crystal changes (ΔF), which is calculated from the recorded frequency values before each nebulisation (baseline frequency, ≈5 MHz) and after deposition of material (≤5 MHz; equation (2)). This ΔF value (Hz) is converted to deposited mass per area (μg/cm^2^) as described in Lenz *et al.* [16].

2 $$ \varDelta F={F}_{baseline}-{F}_{deposited} $$

$$ \varDelta m=\frac{\varDelta F}{56.6} $$

### Anthrone assay

In addition to the QCM, to quantify the deposited mass of CNCs from the ALICE, a colorimetric method for the detection of polysaccharides was adapted from Updegraff *et al*. [30]. This assay also allowed the investigation of the spatial distribution of deposited CNCs. In each well of a 6-well plate, 700 μL of water was exposed to nebulisations of 1 mL of 0.1, 0.5 and 1 mg/mL with 500 μM NaCl of suspended c-CNCs or t-CNCs, respectively. From each well, 500 μL was transferred into sample tubes. Aside, a two-fold dilution series from 0.5 mg/mL to 0.016 mg/mL, including a blank value, were prepared from the stock suspensions. All samples were shaken on ice with 1 mL of 0.1% anthrone reagent (Fluorescent Brightener 28, Merck, Germany) in concentrated sulfuric acid (95–97%, Merck, Germany). After 16 min incubation at 100°C the mixtures were cooled for at least 10 min. Triplicates of 200 μL of each sample were transferred in a flat bottom 96 well plate (TPP Techno Plastic Products AG, Switzerland). The samples were analysed by a plate reader (Benchmark Plus, BioRad, USA) at a 610 nm excitation. The results are represented as mass per area (cm^2^).

### Endotoxin content

Endotoxin content of each CNC and DQ12 suspension was assessed after nebulisation of 0.1, 0.5 and 1 mg/mL of suspended c-CNCs or t-CNCs or 0.1 mg/mL DQ12 upon 1 mL of water within 6-well plate inserts (BD FalconTM Cell Culture Inserts, 3 μm pores, BD USA). Solutions were then collected in sample tubes and quantification of their endotoxin content was performed using the PYROGENTTM - 5000 Limulus Amebocyte Lysate assay (Lonza, USA). A value <0.5 EU/mL for c-CNCs and DQ12 was detected. In the case of t-CNCs the assessed value was 0.9 EU/mL. Endotoxin content of the NP solutions was assessed using the *Limulus Amebocyte* Lysate (LAL) test. All analysis was performed courtesy of Dr. V. Huber at the Institute of Pharmacy, Inselspital, Bern. LFA suspensions have previously been reported to contain non-detectable levels of LAL [5].

### Hazard assessment

#### Lung cell cultures

All *in vitro* exposure experiments were conducted with a three dimensional triple cell co-culture model of the human epithelial airway barrier [52,53] as previously described in detail by Lehmann *et al*. [54] with the addition of CD14^+^ selection, further described by Steiner *et al.* [55]. Briefly, this model is composed of alveolar epithelial cells (A549) grown to confluence on BD Falcon cell culture inserts (high pore density PET membranes, 4.2 cm^2^ growth area, 3.0 μm pore size; Becton Dickinson AG, Switzerland). The model is further supplemented on the apical side with human-derived monocyte-derived macrophages (MDM) as well as with dendritic cells (MDDC) on the baso-lateral side.

### CNC exposures

Triple cell co-cultures were exposed to 1 mL aqueous suspensions of 0.1, 0.5 and 1.0 mg/mL in 500 μM NaCl, as well as a control exposure to NaCl only. Crystalline quartz was further used as a positive particulate aerosol control, specifically Dörentruper Quartz (DQ12; ≤ 5 μm [56]) at 0.1 mg/mL in 500 μM NaCl. DQ12 suspensions were prepared by sonication in the same manner as described for both CNC samples. Additionally, long fibre amosite (LFA) was used in pseudo-ALI exposure experiments as a positive fibrous control to show the sensitivity of the co-culture to fibres *in vitro* [57,58]. The following procedure is defined as pseudo-ALI: a volume of 100 μl of a 0.05 mg/mL stock LFA suspension in supplemented medium was added to the apical compartment of the triple cell co-culture model at the ALI. This methodology was used due to the fact that LFA suspensions are highly unstable and hence unsuitable for nebulisation. Nebulisations in the ALICE should be performed with stable fibre dispersions to account for controllability in all aspects. After exposure, a 24 h post-incubation period at 37°C, 5% CO_2_ followed prior to sampling for biochemical and microscopy analyses. Samples were either removed from the lower compartment of the exposed cells (*i.e*. for cytotoxicity and (pro-)inflammatory response assessment) or in combination with the cells from the insert (investigation of oxidative stress status of the cell cultures) and stored as per the diagnostic kit instructions until analysis could occur.

### Cytotoxicity

To measure cytotoxicity, the release of the intracellular enzyme lactate dehydrogenase (LDH), indicative of cell membrane damage, was assessed by the LDH cytotoxicity detection kit (Roche Applied Science, Mannheim, Germany) according to the manufacturer’s guidelines. The test was conducted in triplicates and evaluated in comparison to the negative control. As a positive control, co-cultures were treated with 100 μl of 0.2% Triton X-100 in H_2_O on the apical side and incubated for 24 h at 37°C, 5% CO_2_.

### Chemokine/Cytokine release

The (pro)-inflammatory response was investigated by quantifying the mediators tumor necrosis factor α (TNF-α) and interleukin 8 (IL-8) using the DuoSet ELISA Development Kit (R&D Systems) according to the manufacturer’s protocol. Lipopolysaccharide (LPS from *Pseudomonas aeruginosa* at 1 μg/mL) applied as 100 μl solution on the apical side of the co-cultures served as the positive control for TNF-α and IL-8 induction.

### Oxidative stress

The total amount of reduced glutathione (GSH) in samples was measured and quantified using the glutathione assay kit (Cayman Chemical Company, Ann Arbor, USA) following the manufacturer’s guidelines. Concentrations of GSH are reported relative to the total amount of protein of each corresponding sample. This was quantified by the Pierce bicinchoninic acid (BCA) Protein Assay kit (Pierce Protein research Products, Thermo Scientific, Rockford, USA) according to the manufacturer’s instructions. Values are presented relative to the negative control.

### Confocal laser scanning microscopy

After the post-incubation of 24 h, triple cell co-cultures were fixed for 15 min with 3% paraformaldehyde in phosphate buffered saline (PBS) at room temperature and then transferred to 0.1 M glycine in PBS for 10 min, or stored at 4°C. To permeabilise the cell membrane, cells were washed three times in PBS and subsequently treated with 0.2% Triton X-100 in PBS for 15 min. The actin cytoskeleton and the DNA of all cells were stained with rhodamine phalloidin (R-415; Molecular Probes, Life Technologies Europe B.V., Zug, Switzerland) in a 1:100 dilution and 4′,6-diamidin-2-phenylindol (DAPI) at 1 μg/mL in 0.3% Triton X-100 in PBS, respectively. For microscopy, membranes were embedded in Glycergel (DAKO Schweiz AG, Baar, Switzerland). Visualisation of the samples was conducted with an inverted Zeiss confocal laser scanning microscope 710 (LSM, Axio Observer.Z1). Representative images (z-stacks) were recorded at 5 independent fields of view for each sample. Images were further processed using the 3D reconstruction software IMARIS (Bitplane AG, Zurich, Switzerland).

### Data analysis

All data is presented as the mean ± standard error of the mean (SEM), deriving from three individual experiments for each material (c-CNCs, t-CNCs) (n = 3) unless otherwise stated. Statistics were conducted using GraphPad Prism 6 (GraphPad Software Inc., La Jolla, USA). Separate two-way ANOVAs were performed comparing effects of concentration and material, with either Dunnett’s (concentration) or Sirdak’s (material) post-*hoc* tests, respectively. Separate one-tailed ratio or non-ratio (TBHP values for GSH data only) paired *t*-tests were also performed for DQ12, LFA and LPS against negative control values. Data was considered significant if *p* < 0.05.

## Additional files

Additional file 1: Figure S1. Digital photographs illustrating the stability of CNC suspensions (1 mg/mL) after 2 weeks at room temperature. (a) c-CNCs, (b) t-CNCs. (I) suspensions by mixing the powder, (II) sonication for 10 min, (III) sonication for 1 h and (IV) sonication for 1 h and addition of 500 μM NaCl. Additional file 2:
**Video file – Nebulisation of t-CNCs with the ALICE system.**
Additional file 3: Table S1. Output rates (s) of the nebuliser depending on material and concentration (n = 13–30 ± SD). Additional file 4: Figure S2. Deposited CNCs. Transmission electron microscopy images of 0.1 and 1 mg/mL nebulised c-CNCs (a, b) and t-CNCs (c, d) deposited on pre-coated, protein rich copper grids exposed in the ALICE. Scale bars represent 1 μm. Additional file 5: Figure S3. Spatial distribution of deposited c-CNCs and t-CNCs after nebulisation. The total deposition within each well of a 6 -well plate referring to the nebulised concentrations of 0.1, 0.5 and 1 mg/mL for c-CNCs (-​-​-) and t-CNCs (^__^), respectively. Data is presented as the sum of each mean of three nebulisations. Transmission electron microscopy images of 0.1 and 1 mg/mL nebulised c-CNCs (a, b) and t-CNCs (c, d) deposited on pre-coated, protein rich copper grids exposed in the ALICE. Scale bars represent 1 μm. Additional file 6: Figure S4. Cytotoxicity of CNCs. Lactate dehydrogenase (LDH) release after exposure to c-CNCs (black) or t-CNCs (grey) to the three test concentrations. The dashed line (-​-​-) represents the level of the negative control. Data is presented as the fold increase relative to the negative control. Additional file 7: Figure S5. Cell morphology following CNC, DQ12 and LFA exposures. Confocal laser scanning microscopy images visualising the actin cytoskeleton (red) and the nuclei (cyan) of the *in vitro* triple cell co-culture model exposed to 0.14 and 0.81 μg/cm^2^ c-CNCs (a, b) or t-CNCs (c, d). Furthermore, cell cultures exposed to 0.23 μg/cm^2^ DQ12 *via* the ALICE (e) and 100 μl of 0.05 mg/mL LFA (f) *via* pseudo-ALI are shown. Yellow arrows indicate cells undergoing mitosis. Scale bars represent 30 μm.

## Abbreviations

HARN: High aspect ratio nanoparticle
CNC: Cellulose nanocrystal
ALICE: Air Liquid Interface Cell Exposure System
DQ12: Dörentrupper Quartz
LFA: Long fibre amosite
TEM: Transmission electron microscopy
DDLS: Depolarised dynamic light scattering
QCM: Quartz crystal microbalance
LDH: Lactate dehydrogenase
GSH: Total reduced glutathione
TNF-α: Tumor necrosis factor-α
IL-8: Interleukin-8
ELISA: Enzyme-linked immunosorbent assay
NADPH: Nicotinamide adenine dinucleotide phosphate
OSHA: Occupational safety and health administration
PEL: Permissive exposure limit

## References

[1] Maynard AD, Baron PA, Foley M, Shvedova AA, Kisin ER, Castranova V (2004). Exposure to carbon nanotube material: Aerosol release during the handling of unrefined single-walled carbon nanotube material. J Toxicol Env Health Pt A.

[2] NIOSH: **Current Intelligence Bulletin 65: Occupational exposure to carbon nanotubes and nanofibres.** Cincinnati, OH: US Department of Health and Human Services, Centers for Disease Control, National Institute for Occupational Safety and Health, DHHS (NIOSH) Publication No 2013–145; 2013.

[3] Donaldson K, Murphy FA, Duffin R, Poland CA (2010). Asbestos, carbon nanotubes and the pleural mesothelium: a review of the hypothesis regarding the role of long fibre retention in the parietal pleura, inflammation and mesothelioma. Particle Fibre Toxicol.

[4] Donaldson K, Poland CA (2009). New insights into nanotubes. Nat Nanotechnol.

[5] Poland CA, Duffin R, Kinloch I, Maynard A, Wallace WAH, Seaton A, Stone V, Brown S, MacNee W, Donaldson K (2008). Carbon nanotubes introduced into the abdominal cavity of mice show asbestos-like pathogenicity in a pilot study. Nat Nanotechnol.

[6] Arora S, Rajwade JM, Paknikar KM (2012). Nanotoxicology and in vitro studies: The need of the hour. Toxicol Appl Pharmacol.

[7] Russell WMS (1959). The Principles of Humane Experimental Technique.

[8] Damoiseaux R, George S, Li M, Pokhrel S, Ji Z, France B, Xia T, Suarez E, Rallo R, Madler L, Cohen Y, Hoek EMV, Nel A (2011). No time to lose-high throughput screening to assess nanomaterial safety. Nanoscale.

[9] Hartung T (2011). From alternative methods to a new toxicology. Eur J Pharm Biopharm.

[10] Mestas J, Hughes CCW (2004). Of mice and not men: differences between mouse and human immunology. J Immunol.

[11] Rothen-Rutishauser B, Müller L, Blank F, Brandenberger C, Mühlfeld C, Gehr P (2008). A newly developed in vitro model of the human epithelial airway barrier to study the toxic potential of nanoparticles. ALTEX.

[12] Paur HR, Cassee FR, Teeguarden J, Fissan H, Diabate S, Aufderheide M, Kreyling WG, Hanninen O, Kasper G, Riediker M, Rothen-Rutishauser B, Schmid O (2011). In-vitro cell exposure studies for the assessment of nanoparticle toxicity in the lung-A dialog between aerosol science and biology. J Aerosol Sci.

[13] Maiorano G, Sabella S, Sorce B, Brunetti V, Malvindi MA, Cingolani R, Pompa PP (2010). Effects of cell culture media on the dynamic formation of protein-nanoparticle complexes and influence on the cellular response. ACS Nano.

[14] Brandenberger C, Rothen-Rutishauser B, Muhlfeld C, Schmid O, Ferron GA, Maier KL, Gehr P, Lenz AG (2010). Effects and uptake of gold nanoparticles deposited at the air-liquid interface of a human epithelial airway model. Toxicol Appl Pharmacol.

[15] Fröhlich E, Bonstingl G, Hofler A, Meindl C, Leitinger G, Pieber TR, Roblegg E (2013). Comparison of two in vitro systems to assess cellular effects of nanoparticles-containing aerosols. Toxicology In Vitro.

[16] Lenz AG, Karg E, Lentner B, Dittrich V, Brandenberger C, Rothen-Rutishauser B, Schulz H, Ferron GA, Schmid O (2009). A dose-controlled system for air-liquid interface cell exposure and application to zinc oxide nanoparticles. Particle Fibre Toxicol.

[17] Raemy DO, Grass RN, Stark WJ, Schumacher CM, Clift MJD, Gehr P, Rothen-Rutishauser B (2012). Effects of flame made zinc oxide particles in human lung cells - a comparison of aerosol and suspension exposures. Particle Fibre Toxicol.

[18] Creutzenberg O (2012). Biological interactions and toxicity of nanomaterials in the respiratory tract and various approaches of aerosol generation for toxicity testing. Arch Toxicol.

[19] Kasai T, Gotoh K, Nishizawa T, Sasaki T, Katagiri T, Umeda Y, Toya T, Fukushima S (2014). Development of a new multi-walled carbon nanotube (MWCNT) aerosol generation and exposure system and confirmation of suitability for conducting a single-exposure inhalation study of MWCNT in rats. Nanotoxicology.

[20] Ma-Hock L, Treumann S, Strauss V, Brill S, Luizi F, Mertler M, Wiench K, Gamer AO, van Ravenzwaay B, Landsiedel R (2009). Inhalation toxicity of multiwall carbon nanotubes in rats exposed for 3 months. Toxicol Sci.

[21] McKinney W, Chen B, Frazer D (2009). Computer controlled multi-walled carbon nanotube inhalation exposure system. Inhal Toxicol.

[22] Porter DW, Hubbs AF, Chen BT, McKinney W, Mercer RR, Wolfarth MG, Battelli L, Wu N, Sriram K, Leonard S, Andrew M, Willard P, Tsuruoka S, Endo M, Tsukada T, Munekane F, Frazer DG, Castranova V (2013). Acute pulmonary dose-responses to inhaled multi-walled carbon nanotubes. Nanotoxicology.

[23] Sayes CM, Reed KL, Glover KP, Swain KA, Ostraat ML, Donner EM, Warheit DB (2010). Changing the dose metric for inhalation toxicity studies: Short-term study in rats with engineered aerosolized amorphous silica nanoparticles. Inhal Toxicol.

[24] Shvedova AA, Kisin E, Murray AR, Johnson VJ, Gorelik O, Arepalli S, Hubbs AF, Mercer RR, Keohavong P, Sussman N, Jin J, Yin J, Stone S, Chen BT, Deye G, Maynard A, Castranova V, Baron PA, Kagan VE (2008). Inhalation vs. aspiration of single-walled carbon nanotubes in C57BL/6 mice: inflammation, fibrosis, oxidative stress, and mutagenesis. Am J Physiol Lung Cell Mol Physiol.

[25] Eichhorn SJ, Dufresne A, Aranguren M, Marcovich NE, Capadona JR, Rowan SJ, Weder C, Thielemans W, Roman M, Renneckar S, Gindl W, Veigel S, Keckes J, Yano H, Abe K, Nogi M, Nakagaito AN, Mangalam A, Simonsen J, Benight AS, Bismarck A, Berglund LA, Peijs T (2010). Review: current international research into cellulose nanofibres and nanocomposites. J Mater Sci Mater.

[26] Moon RJ, Martini A, Nairn J, Simonsen J, Youngblood J (2011). Cellulose nanomaterials review: structure, properties and nanocomposites. Chem Soc Rev.

[27] Ferguson W (2012). Wood pulp is surprise new wonder material. New Scientist.

[28] Azizi Samir MAS, Alloin F, Dufresne A (2005). Review of recent research into cellulosic whiskers, their properties and their application in nanocomposite field. Biomacromolecules.

[29] De Souza Lima MM, Wong JT, Paillet M, Borsali R, Pecora R (2003). Translational and rotational dynamics of rodlike cellulose whiskers. Langmuir.

[30] Updegraff DM (1969). Semimicro determination of cellulose in biological materials. Anal Biochem.

[31] Clift MJD, Boyles MSP, Brown DM, Stone V (2010). An investigation into the potential for different surface-coated quantum dots to cause oxidative stress and affect macrophage cell signalling in vitro. Nanotoxicology.

[32] Dong XM, Kimura T, Revol J-F, Gray DG (1996). Effects of ionic strength on the isotropic − chiral nematic phase transition of suspensions of cellulose crystallites. Langmuir.

[33] Jorfi M, Roberts MN, Foster EJ, Weder C (2013). Physiologically responsive, mechanically adaptive bio-nanocomposites for biomedical applications. ACS Appl Mater Interfaces.

[34] Shanmuganathan K, Capadona JR, Rowan SJ, Weder C (2010). Stimuli-responsive mechanically adaptive polymer nanocomposites. ACS Appl Mater Interfaces.

[35] Bouwmeester H, Lynch I, Marvin HJP, Dawson KA, Berges M, Braguer D, Byrne HJ, Casey A, Chambers G, Clift MJD, Elia G, Fernandes TF, Fjellsbø LB, Hatto P, Juillerat L, Klein C, Kreyling WG, Nickel C, Riediker M, Stone V (2011). Minimal analytical characterization of engineered nanomaterials needed for hazard assessment in biological matrices. Nanotoxicology.

[36] Kovacs T, Naish V, O’Connor B, Blaise C, Gagne F, Hall L, Trudeau V, Martel P (2010). An ecotoxicological characterization of nanocrystalline cellulose (NCC). Nanotoxicology.

[37] Pereira MM, Raposo NRB, Brayner R, Teixeira EM, Oliveira V, Quintão CCR, Camargo LSA, Mattoso LHC, Brandão HM (2013). Cytotoxicity and expression of genes involved in the cellular stress response and apoptosis in mammalian fibroblast exposed to cotton cellulose nanofibers. Nanotechnology.

[38] *Occupational Safety and Health Standards. Cotton dust.* ;[https://www.osha.gov/pls/oshaweb/owadisp.show_document?p_id=10053&p_table=STANDARDS]

[39] Wang X, Yong ZZ, Li QW, Bradford PD, Liu W, Tucker DS, Cai W, Wang H, Yuan FG, Zhu YT (2012). Ultrastrong, stiff and multifunctional carbon nanotube composites. Materials Res Lett.

[40] **WHO report of the International programme on chemical safety (IPCS); Environmental health criteria 53: Asbestos and other natural mineral fibers.**[http://www.inchem.org/documents/ehc/ehc/ehc53.htm]

[41] Clift MJD, Foster EJ, Vanhecke D, Studer D, Wick P, Gehr P, Rothen-Rutishauser B, Weder C (2011). Investigating the interaction of cellulose nanofibers derived from cotton with a sophisticated 3D human lung cell coculture. Biomacromolecules.

[42] Bruch J, Rehn S, Rehn B, Borm PJA, Fubini B (2004). Variation of biological responses to different respirable quartz flours determined by a vector model. Int J Hyg Environ Health.

[43] Clouter A, Brown D, Höhr D, Borm P, Donaldson K (2001). Inflammatory effects of respirable quartz collected in workplaces versus standard DQ12 quartz: particle surface correlates. Toxicol Sci.

[44] Monteiller C, Tran L, MacNee W, Faux S, Jones A, Miller B, Donaldson K (2007). The pro-inflammatory effects of low-toxicity low-solubility particles, nanoparticles and fine particles, on epithelial cells in vitro: the role of surface area. Occup Environ Med.

[45] Davis J, Addison J, Bolton R, Donaldson K, Jones A, Smith T (1986). The pathogenicity of long versus short fibre samples of amosite asbestos administered to rats by inhalation and intraperitoneal injection. Br J Exp Pathol.

[46] Donaldson K, Brown G, Brown D, Bolton R, Davis J (1989). Inflammation generating potential of long and short fibre amosite asbestos samples. Br J Ind Med.

[47] Donaldson K, Poland CA, Schins RPF (2010). Possible genotoxic mechanisms of nanoparticles: Criteria for improved test strategies. Nanotoxicology.

[48] Capadona JR, Van Den Berg O, Capadona LA, Schroeter M, Rowan SJ, Tyler DJ, Weder C (2007). A versatile approach for the processing of polymer nanocomposites with self-assembled nanofibre templates. Nat Nano.

[49] Camarero Espinosa S, Kuhnt T, Foster EJ, Weder C (2013). Isolation of thermally stable cellulose nanocrystals by phosphoric acid hydrolysis. Biomacromolecules.

[50] Schneider CA, Rasband WS, Eliceiri KW (2012). NIH Image to ImageJ: 25 years of image analysis. Nat Meth.

[51] Endes C, Müller S, Schmid O, Vanhecke D, Foster EJ, Petri-Fink A, Rothen-Rutishauser B, Weder C, Clift MJD (2013). Risk assessment of released cellulose nanocrystals – mimicking inhalatory exposure. J Phys Conf Ser.

[52] Rothen-Rutishauser BM, Kiama SG, Gehr P (2005). A three-dimensional cellular model of the human respiratory tract to study the interaction with particles. Am J Respir Cell Mol Biol.

[53] Blank F, Rothen-Rutishauser BM, Schurch S, Gehr P (2006). An optimized in vitro model of the respiratory tract wall to study particle cell interactions. J Aerosol Med.

[54] Lehmann A, Brandenberger C, Blank F, Gehr P, Rothen-Rutishauser B: **A 3D model of the human epithelial airway barrier**. In *Alternatives to animal testing.* Edited by ML Y, RS L: Artech House; 2010:239–260.

[55] Steiner S, Mueller L, Popovicheva OB, Raemy DO, Czerwinski J, Comte P, Mayer A, Gehr P, Rothen-Rutishauser B, Clift MJD (2012). Cerium dioxide nanoparticles can interfere with the associated cellular mechanistic response to diesel exhaust exposure. Toxicol Lett.

[56] Robock K (1973). Standard Quartz DQ12 < 5 μm for experimental pneumoconiosis research projects in the Federal Republic of Germany. Ann Occup Hyg.

[57] Rusli R, Eichhorn SJ (2008). Determination of the stiffness of cellulose nanowhiskers and the fiber-matrix interface in a nanocomposite using Raman spectroscopy. Appl Phys Lett.

[58] Šturcová A, Davies GR, Eichhorn SJ (2005). Elastic modulus and stress-transfer properties of tunicate cellulose whiskers. Biomacromolecules.

