# Supplementary figures and images for: An in vitro testing strategy towards mimicking the inhalation of high aspect ratio nanoparticles
Source: Part Fibre Toxicol. 2014 Sep 23;11:40. doi: 10.1186/s12989-014-0040-x (PMC4189630; doi:10.1186/s12989-014-0040-x)

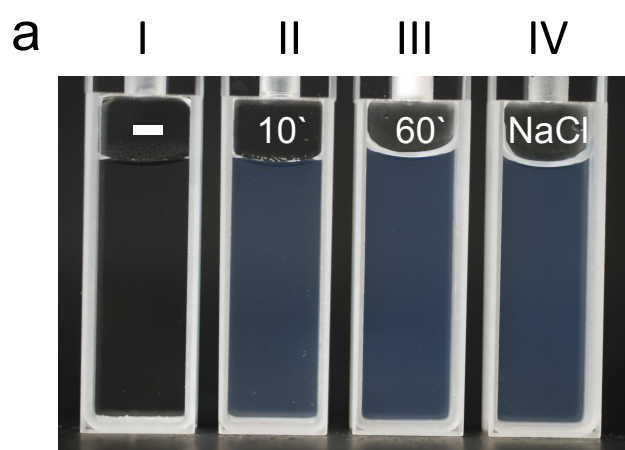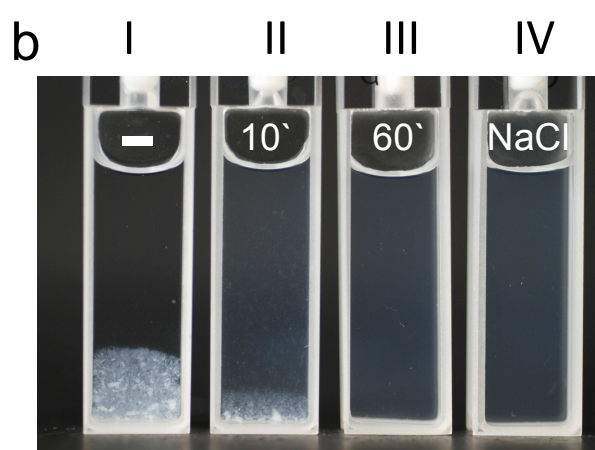

Supplement: Additional file 1: Figure S1. — Digital photographs illustrating the stability of CNC suspensions (1 mg/mL) after 2 weeks at room temperature. (a) c-CNCs, (b) t-CNCs. (I) suspensions by mixing the powder, (II) sonication for 10 min, (III) sonication for 1 h and (IV) sonication for 1 h and addition of 500 μM NaCl. [file 12989_2014_40_MOESM1_ESM.pdf]

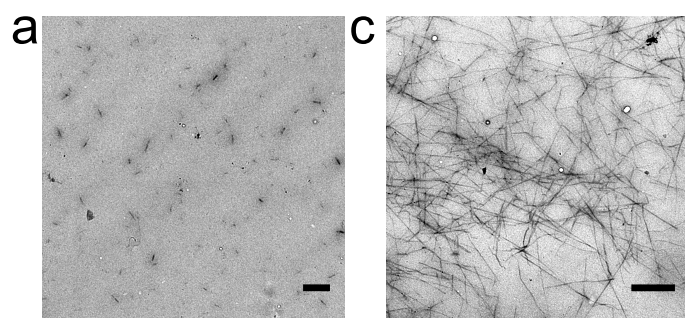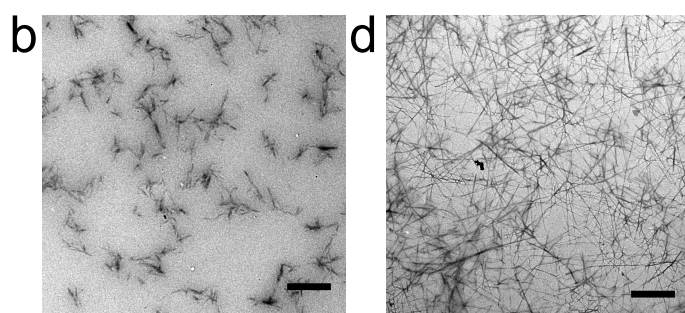

Supplement: Additional file 4: Figure S2. — Deposited CNCs. Transmission electron microscopy images of 0.1 and 1 mg/mL nebulised c-CNCs (a, b) and t-CNCs (c, d) deposited on pre-coated, protein rich copper grids exposed in the ALICE. Scale bars represent 1 μm. [file 12989_2014_40_MOESM4_ESM.pdf]

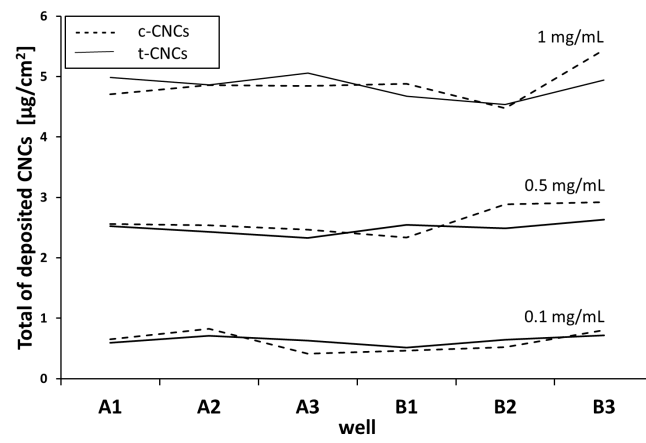

Supplement: Additional file 5: Figure S3. — Spatial distribution of deposited c-CNCs and t-CNCs after nebulisation. The total deposition within each well of a 6 -well plate referring to the nebulised concentrations of 0.1, 0.5 and 1 mg/mL for c-CNCs (-​-​-) and t-CNCs (__), respectively. Data is presented as the sum of each mean of three nebulisations. Transmission electron microscopy images of 0.1 and 1 mg/mL nebulised c-CNCs (a, b) and t-CNCs (c, d) deposited on pre-coated, protein rich copper grids exposed in the ALICE. Scale bars represent 1 μm. [file 12989_2014_40_MOESM5_ESM.pdf]

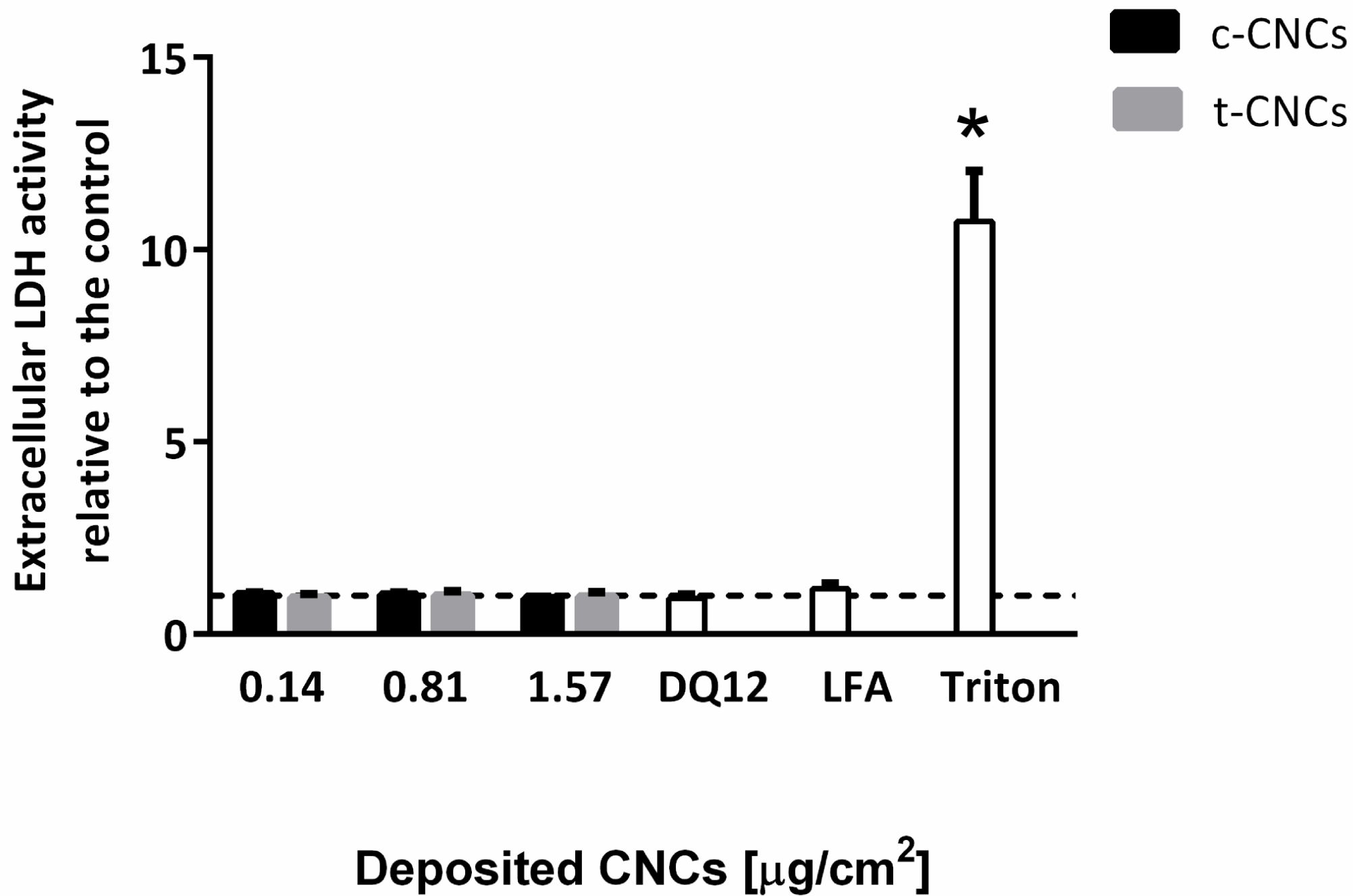

Supplement: Additional file 6: Figure S4. — Cytotoxicity of CNCs. Lactate dehydrogenase (LDH) release after exposure to c-CNCs (black) or t-CNCs (grey) to the three test concentrations. The dashed line (-​-​-) represents the level of the negative control. Data is presented as the fold increase relative to the negative control. [file 12989_2014_40_MOESM6_ESM.pdf]

5

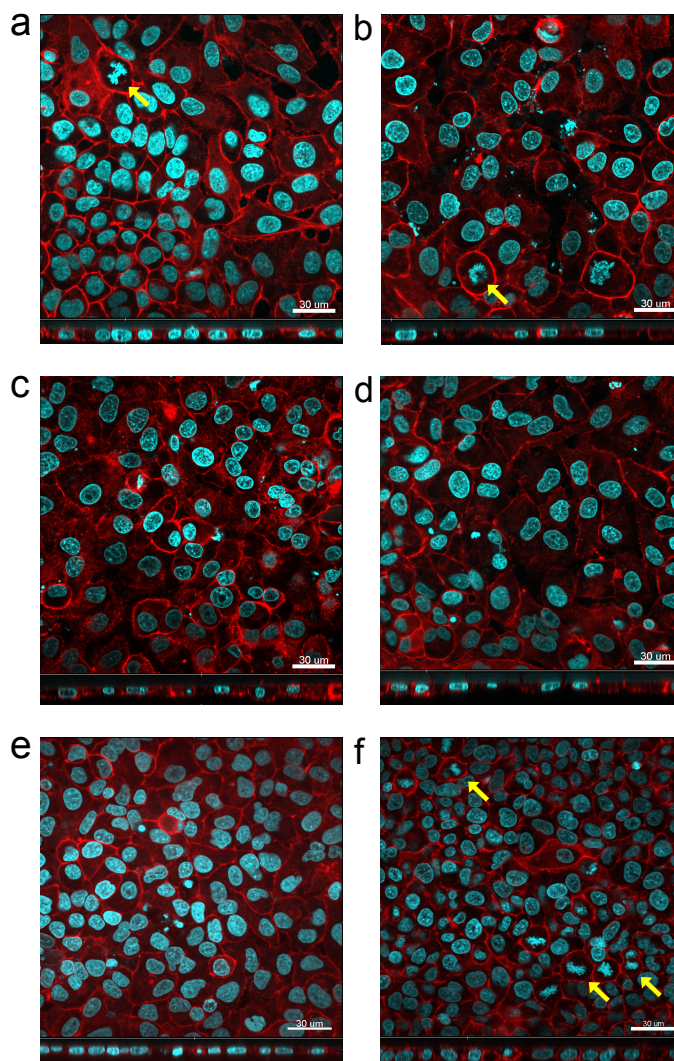

Supplement: Additional file 7: Figure S5. — Cell morphology following CNC, DQ12 and LFA exposures. Confocal laser scanning microscopy images visualising the actin cytoskeleton (red) and the nuclei (cyan) of the in vitro triple cell co-culture model exposed to 0.14 and 0.81 μg/cm2 c-CNCs (a, b) or t-CNCs (c, d). Furthermore, cell cultures exposed to 0.23 μg/cm2 DQ12 via the ALICE (e) and 100 μl of 0.05 mg/mL LFA (f) via pseudo-ALI are shown. Yellow arrows indicate cells undergoing mitosis. Scale bars represent 30 μm. [file 12989_2014_40_MOESM7_ESM.pdf]
