# Supplementary material for: An in vitro testing strategy towards mimicking the inhalation of high aspect ratio nanoparticles
Source: Part Fibre Toxicol. 2014 Sep 23;11:40. doi: 10.1186/s12989-014-0040-x (PMC4189630; doi:10.1186/s12989-014-0040-x)
Supplement: Additional file 3: Table S1. — Output rates (s) of the nebuliser depending on material and concentration (n = 13–30 ± SD). [file 12989_2014_40_MOESM3_ESM.doc]

**Additional file 3: Table S1 Output rates (s) of the nebuliser depending on material and concentration (n= 13-30 ±SD).**

| **mg/mL** | **0** | **0.1** | **0.5** | **1.0** |
| --- | --- | --- | --- | --- |
| **c-CNC** | - | 100±19 | 139±63 | 134±89 |
| **t-CNC** | - | 93±29 | 127±37 | 175±61 |
| **NaCl** | 82±29 | - | - | - |
